# MRI morphological and functional method for clear distinction of patients with left ventricular non-compaction, inflammatory dilated cardiomyopathy and physiological myocardial trabeculation

**DOI:** 10.1186/1532-429X-14-S1-M7

**Published:** 2012-02-01

**Authors:** Astrid Burger, Stephanie Lehrke, Andreas Voss, Hugo A Katus, Henning Steen

**Affiliations:** 1 Department of Cardiology, University of Heidelberg, Heidelberg, Germany; 2 Institute of Psychology, University of Heidelberg, Heidelberg, Germany

## Background

Left ventricular non-compaction (LVNC) cardiomyopathy is characterized by a thin, compacted epimyocardial and a thick non-compacted, trabeculated endomyocardial layer. High-resolution cardiac magnetic resonance imaging (CMR) has been clinically successfully used to establish a Non-compact-to-compact- (NCTC) ratio of 2.3 in a 4-chamber view which is regarded pathological. Clinically, in patients with dilated cardiomyopathy (DCMP) and noticeably even in volunteers with physiological myocardial trabecularisation, up to 28% of verifiably healthy participants demonstrated the NCTC-ratio ≥ 2.3 so that there is a clinical need for a better distinction between patients with LVNC, DCMP and healthy subjects.

We hypothesized that MRI is able to clearly distinguish LVNC from DCMP and healthy volunteers by a novel combined MRI and statistical approach of morphological and functional parameters.

## Methods

**Figure 1:**
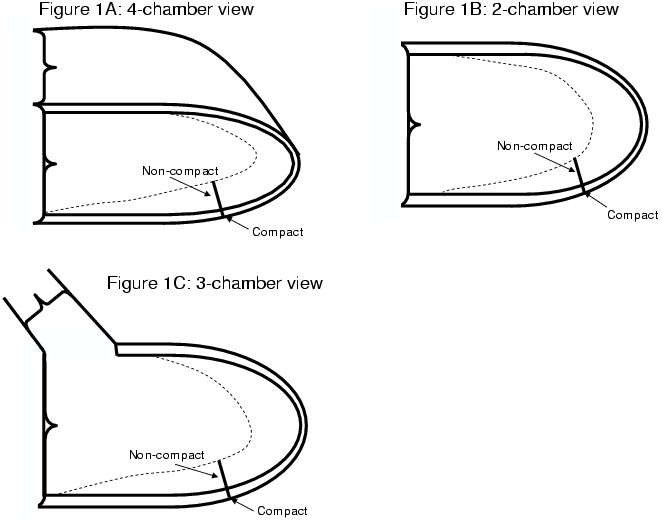


31 LVNC patients, defined by an MRI NCTC-ratio >2.3 (in a 4-chamber view plus one additional parameter (fulfilment of echo-criteria, histology, genetic proof, invasive X-ray), 13 patients with histologically proven inflammatory DCMP as well as 117 male/female healthy volunteers were studied employing a vector-ECG gated multi-slice 2-, 3-, 4-chamber and short axis (SA) standard cine SSFP-sequence covering the entire left ventricle. Functional parameters like end-diastolic, end-systolic volumes and ejection fraction were generated via SA SSFP slices as usual. Compacted and non-compacted thicknesses were defined in 2-,3-,4-chamber views as illustrated in Figure 1A-C, as previously published. With a stepwise discriminant analysis, statistically significant morphological and functional predictors for clear distinction of LVNC, DCMP and physiological trabeculation were found and were successfully cross-validated with a jackknife procedure.

## Results

**Figure 2:**
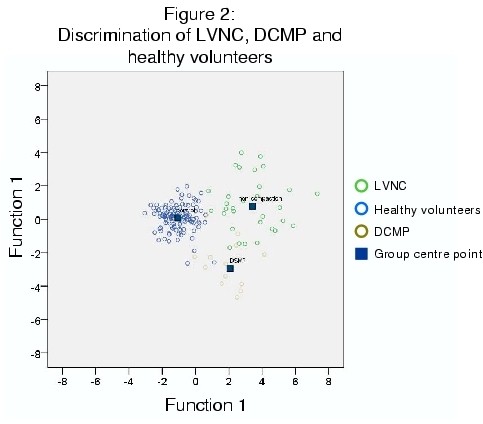


Six predictors were determined to improve correct prediction: ejection fraction, compact thicknesses in 2- and 4-chamber views, NCTC-ratios in 2-and 4-chamber views and the non-compacted thickness in 3-chamber views. The combination of these six predictors led to a correct categorisation in 93% of cases which can be seen from Figure 2.

## Conclusions

The correct identification of patients with cardiomyopathies and their clear distinction from healthy volunteers is clinically and therapeutically as well as socio-economically of paramount importance. By simple combination of values for ejection fraction, compact thicknesses in 2- and 4-chamber views, NCTC-ratios in 2-and 4-chamber views and the non-compacted thickness in 3-chamber views, we present a method that sufficiently distincts LVNC and DCMP patients from healthy volunteers. Prospective clinical trials have to be conducted to validate this approach in the future.

## Funding

None.

